# Mixed pathologies including chronic traumatic encephalopathy account for dementia in retired association football (soccer) players

**DOI:** 10.1007/s00401-017-1680-3

**Published:** 2017-02-15

**Authors:** Helen Ling, Huw R. Morris, James W. Neal, Andrew J. Lees, John Hardy, Janice L. Holton, Tamas Revesz, David D. R. Williams

**Affiliations:** 1 Queen Square Brain Bank for Neurological Disorders, UCL Institute of Neurology, University College London, London, UK; 2 Reta Lila Weston Institute for Neurological Studies, UCL Institute of Neurology, 1 Wakefield Street, WC1N 1PJ London, UK; 3 Department of Molecular Neuroscience, UCL Institute of Neurology, University College London, London, UK; 4 Department of Clinical Neuroscience, UCL Institute of Neurology, London, UK; 5 Department of Cellular Pathology, Cardiff University, Cardiff, Wales, UK; 6 Cefn Coed Hospital, Swansea, Wales, UK

**Keywords:** Chronic traumatic encephalopathy, Soccer, Football, Heading, Traumatic brain injury, Concussion, Tauopathy

## Abstract

In retired professional association football (soccer) players with a past history of repetitive head impacts, chronic traumatic encephalopathy (CTE) is a potential neurodegenerative cause of dementia and motor impairments. From 1980 to 2010, 14 retired footballers with dementia were followed up regularly until death. Their clinical data, playing career, and concussion history were prospectively collected. Next-of-kin provided consent for six to have post-mortem brain examination. Of the 14 male participants, 13 were professional and 1 was a committed amateur. All were skilled headers of the ball and had played football for an average of 26 years. Concussion rate was limited in six cases to one episode each during their careers. All cases developed progressive cognitive impairment with an average age at onset of 63.6 years and disease duration of 10 years. Neuropathological examination revealed septal abnormalities in all six post-mortem cases, supportive of a history of chronic repetitive head impacts. Four cases had pathologically confirmed CTE; concomitant pathologies included Alzheimer’s disease (*N* = 6), TDP-43 (*N* = 6), cerebral amyloid angiopathy (*N* = 5), hippocampal sclerosis (*N* = 2), corticobasal degeneration (*N* = 1), dementia with Lewy bodies (*N* = 1), and vascular pathology (*N* = 1); and all would have contributed synergistically to the clinical manifestations. The pathological diagnosis of CTE was established in four individuals according to the latest consensus diagnostic criteria. This finding is probably related to their past prolonged exposure to repetitive head impacts from head-to-player collisions and heading the ball thousands of time throughout their careers. Alzheimer’s disease and TDP-43 pathologies are common concomitant findings in CTE, both of which are increasingly considered as part of the CTE pathological entity in older individuals. Association football is the most popular sport in the world and the potential link between repetitive head impacts from playing football and CTE as indicated from our findings is of considerable public health interest. Clearly, a definitive link cannot be established in this clinico-pathological series, but our findings support the need for further systematic investigation, including large-scale case–control studies to identify at risk groups of footballers which will justify for the implementation of protective strategies.

## Introduction

First reported as ‘punch drunk syndrome’ in boxers, the long-term neurodegenerative consequence of repetitive mild traumatic brain injury (rTBI) is now known as chronic traumatic encephalopathy (CTE) [32]. CTE has since been reported in a range of contact sports, most notably in American football [31]. The clinical features of CTE are variable and consist of a combination of mood and behavioural changes, memory loss, executive dysfunction, slurred speech, parkinsonism, and gait impairment, which typically manifest years after the initial rTBI [24, 32]. In some cases, the clinical presentations may be indistinguishable from frontotemporal dementia (FTD), Alzheimer’s disease (AD), atypical parkinsonism, and cerebellar ataxia [40]. While dementia in older individuals is commonly caused by mixed pathologies in particular vascular and neurodegenerative diseases [22], in those with a history of rTBI, CTE is an additional differential diagnosis, which, at present, can only be confirmed by neuropathological examination due to the lack of validated clinical diagnostic criteria [30].

Heading the ball is an integral part of association football (known as soccer in North America) and may produce considerable repetitive impacts to the head [36]. An average player heads the ball 6–12 times per game and performs at least 2000 headers during a 20-year career in addition to repetitive heading drills at training [36]. Head injuries in football are nevertheless more frequently caused by head-player contact (40%, including head, arm, and leg) than head-ball contact (12.6%, including accidental heading) [5]. Many head impacts in football do not result in concussion and overt neurological symptoms [3, 19, 36], yet are associated with subtle neuropsychiatric deficits or changes in functional MRI, and are referred as ‘subconcussion’ [31]. Brain structural and cognitive changes have been reported in footballers exposed to repetitive subconcussive head impacts including heading [20, 21, 26, 28, 36, 42, 43]. Since repetitive head impacts can be substantial in football [19], their clinical significance as a potential cause of subclinical TBI and a risk of later development of CTE are of considerable public health interest.

Dementia as a potential late life consequence of playing professional football attracted media attention following the death of the 59-year-old West Bromwich Albion centre-forward, Jeff Astle, who had a 5-year history of progressive cognitive decline and, more recently, lay-press reports of dementia in four of the eight surviving footballers in the 1966 England’s World Cup winning team. The pathological substrates of dementia syndrome in retired footballers remain elusive with only four post-mortem reports of footballers in the literature [4, 13, 14, 31]. In this study, we describe the clinical and pathological features of a group of retired professional footballers who developed dementia in later life.

## Methods

Informed consent to participate in this study was obtained from each participant or their designated next-of-kin during life. The next-of-kin or close surviving relatives provided written consent to the publication of the clinical and pathological data included in this article. This study was conducted at the Queen Square Brain Bank for Neurological Studies (QSBB), UCL Institute of Neurology, under the ethics approval granted by the London Ethics Committee (REC Reference: 02/N093).

### Participants

**Fig. 1:**
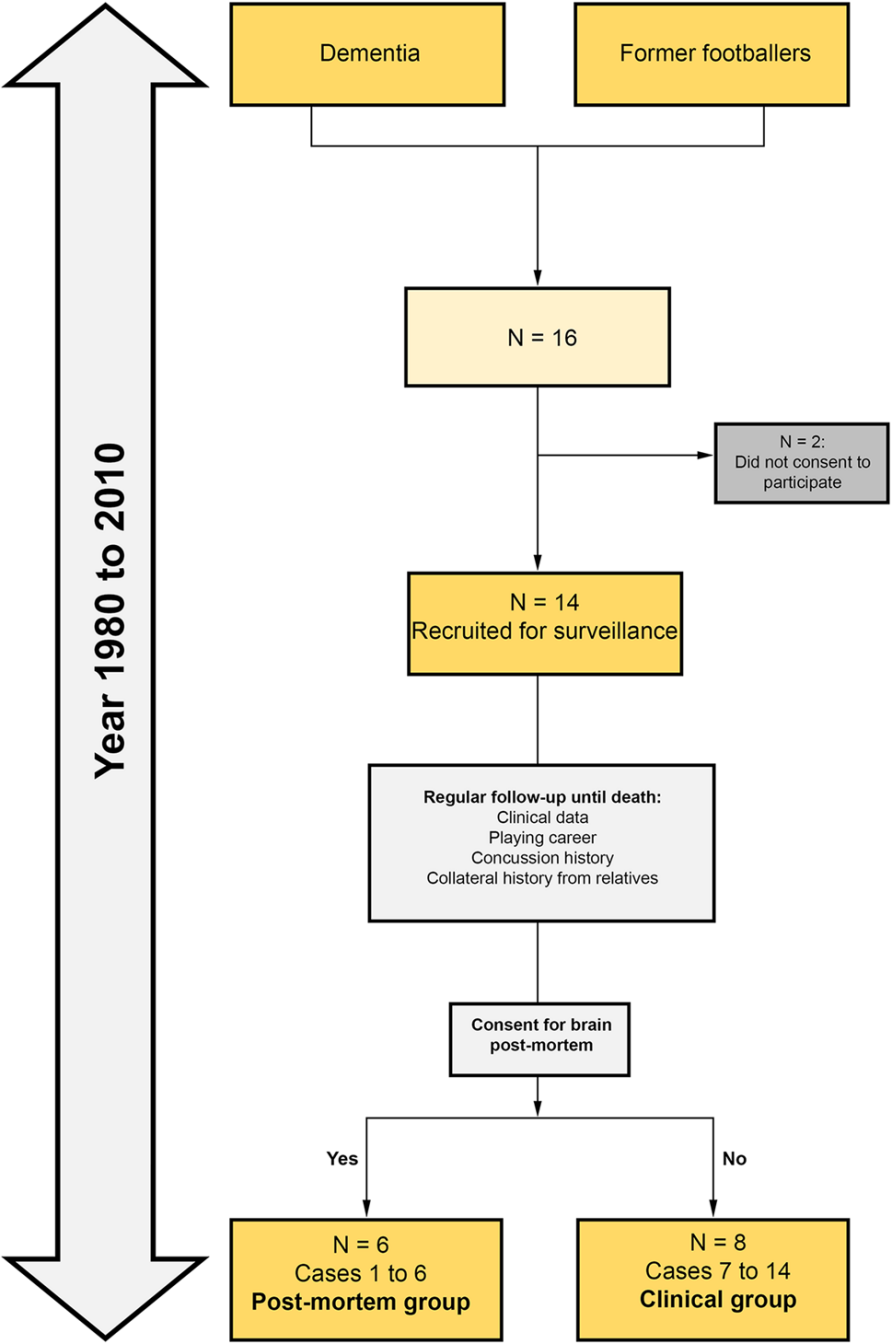
Flow diagram illustrating the number of cases included in the clinical and post-mortem groups of ex-footballers with dementia

Between 1980 and 2010, 16 consecutive cases of retired footballers with progressive cognitive impairment were identified at the Old Age Psychiatry Service in Swansea, Wales, UK, and were enrolled for clinical surveillance and regular out-patient follow-up by a consultant psychiatrist (DDRW) until death (Fig. 1). Next-of-kin consented for 14 to be included in the surveillance and for six to have post-mortem brain examination. Collateral history, playing career, and concussion history from close relatives were prospectively collected. From 2015 to 2016, clinical and other demographic data were systematically and retrospectively collected from review of medical records and interviews of close relatives for the present clinico-pathological series.

### Neuropathological methods and diagnoses

Neuropathological examination of the brain was initially performed at the Department of Cellular Pathology, Cardiff University (JWN) between year 2005 and 2009. All six post-mortem cases had received an original pathological diagnosis of AD. In 2016, tissue blocks of these six cases were transferred to the Queen Square Brain Bank, UCL Institute of Neurology, London, UK for systemic neuropathological analysis for the present study. Eight-µm-thick histological sections were stained using routine histological (haematoxylin and eosin, H&E) technique. Immunohistochemistry using modern antibodies to the following proteins was performed using a standard avidin–biotin method: tau (AT8 clone; Thermo scientific MN1020; 1:600), 3-repeat tau (Gift from Dr Rohan de Silva; 1:150) and 4-repeat tau (Gift from Dr Rohan de Silva; 1:750), amyloid-β (Aβ; Biosource international, Mouse Dako, clone 6F/3D; 1:100), transactive response DNA-binding protein 43 kDa (TDP-43; monoclonal; clone 2E2-D3; 1:2000), p62 (BD Transduction Labs; 1:200), α-synuclein (Novocastra; 1:50), Iba-1 (Wako; polyclonal rabbit 1:1000), and SMI-31 (Biolegend; monoclonal mouse 1:1000).

A consensus agreement regarding the histological features of CTE was reached among two neuropathologists (TR, JLH), following the recently published NINDS diagnostic criteria [30]. The finding of hyperphosphorylated tau accumulation in neurons, astrocytes, and cell processes around small blood vessels with predilection to the cortical sulci is mandatory for the diagnosis of CTE [12], which is distinct from AD [15], primary age-related tauopathy (PART) [10], and age-related tau astrogliopathy (ARTAG) [23]. Supportive features of CTE were assessed [30]. Other neuropathological diagnoses were made following consensus criteria for AD according to the National Institute on Aging-Alzheimer’s Association (NIA-AA) Guidelines [16], Lewy body disease (LBD) [7, 33], and corticobasal degeneration (CBD) [11].

Brain regions evaluated are summarised in Table 1. Briefly, H&E sections were used to assess for hippocampal sclerosis, neuronal loss in the substantia nigra, and vascular pathology in the frontal, temporal and parietal regions, striatum, pons, and cerebellum. CTE-type pathologies were determined by screening the AT8 sections of the following brain regions: frontal, temporal, and parietal cortices, hippocampus, amygdala, basal ganglia, midbrain, pons, medulla, and cerebellum. Five consecutive 8-µm-thick sections were prepared from tissue blocks of the frontal, temporal, and parietal cortices to screen for the pathognomonic hyperphosphorylated tau accumulation in the cortical sulci [30]. The presence of the following additional pathologies was systematically assessed: TDP-43 proteinopathy (TDP-43 lesions in frontal, temporal, and parietal cortices, hippocampus, amygdala, basal ganglia, and midbrain), argyrophilic grain disease [35] (AT8 and p62 immunohistochemical preparations in amygdala and hippocampus), *C9orf72* expansion-specific p62-positive neuronal cytoplasmic inclusions (p62 immunohistochemical preparation in the hippocampus and cerebellum), Lewy body pathologies using α-synuclein immunohistochemistry (amygdala, midbrain, and pons), AD-related Aβ pathologies for Thal phase score [41], and cerebral amyloid angiopathy (Aβ immunohistochemistry in the frontal, temporal, and parietal cortices, and cerebellum). To determine the level of Alzheimer’s disease neuropathological change, ABC score was established according to the NIA-AA guidelines [16]. Tissue sections of frontal white matter, internal capsule, and cerebellar white matter were stained with the SMI-31 antibody and reviewed for evidence of diffuse axonal injury, except in Case 5, in which the SMI-31 section of the frontal cortex was unavailable. Iba1 immunohistochemistry was performed to assess microglia/macrophage response, except in Case 5, in which the IBA1 section of the frontal cortex was unavailable.

**Table 1:**
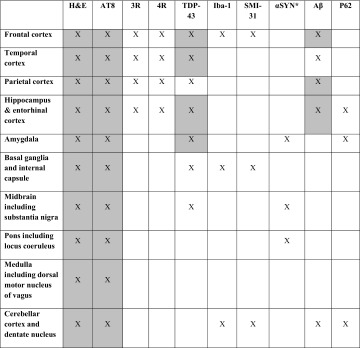
Brain regions evaluated in the six post-mortem cases

Grey boxes represent the sampling regions recommended by the preliminary NINDS criteria for the neuropathological diagnosis of CTE [30]

*H&E* haematoxylin and eosin; antibodies for immunohistochemistry, *3R* 3-repeat tau, *4R* 4-repeat tau, *αSYN* alpha-synuclein, *Aβ* beta-amyloid, *AT8* tau, *Iba-1* microglia, *p62* for argyrophilic grains in amygdala and hippocampus and *C9orf72* inclusions in hippocampus and amygdala, *SMI-31* phosphorylated neurofilament, *TDP-43* transactive response DNA-binding protein, 43 kDa

^a^If αSYN is positive in midbrain, pons and amygdala, then including frontal and temporal cortices and hippocampus

### Statistical analysis

The SPSS 24.0 statistical package was used. Student’s *t* test was used to compare continuous data.

## Results

All 14 retired footballers were male, 13 were professional, and 1 was a committed amateur (Case 4). They were all recognised as skilled headers of the ball, with half of them playing in centre-half or centre-forward positions, where heading of the ball is frequent (Table 2). They all began playing football regularly in their childhood or early teens for an average of 26 years. Concussion was only reported in six footballers, limited to a single episode in each during their careers. Two participants also boxed as amateurs, one of whom served in the military, but none reported any episodes of concussion during these activities.

**Table 2:** Summary of demographic and clinical data of 14 retired footballers with dementia (Cases 1–14)

Case	Age at death	Neuropathological examination	Years playing football	Football playing position	Participation in other sports	Military service	History of concussion (No. of episode)	History of seizure	Family history	Age at symptom onset	Disease duration (years)	Presenting symptoms	Progressive dementia^a^	Recurrent hallucination	Behavioural changes	Mood changes	Motor impairment	Final clinical Diagnosis
1	65	Yes	36	Centre-forward	–	–	–	–	–	56	9	Anx, dep, Dysar, G	Yes	–	Exp, Imp, Agg	Apa, Dep	Park, G, PI, Dysar	FTD and PD
2	78	Yes	20	Fullback	–	–	Head-to-head collision with LOC and skull fracture (1)	–	Sister (Dementia)	69	9	Exp, Imp, M	Yes	–	Exp, Imp, Agg	Apa, Dep	–	AD and VasD
3	74	Yes	30	Centre-half	Cricket	–	Head-to-head collision with LOC (1)	Occasional GTC seizures in advanced disease	–	66	8	M	Yes	–	Exp, Imp, Agg	Apa	–	AD
4	60	Yes	23	Centre-forward	Amateur boxing	–	Mid-air head-to-player collision with LOC (1)	–	–	55	5	Agg, M	Yes	Yes	Exp, Imp, Agg	Apa, Dep	Park, G, Dysph	AD and PD
5	72	Yes	25	Wing-half	–	–	Collison with fractured jaw but no LOC (1)	–	–	63	9	Agg, Aph, Imp, M	Yes	Yes	Exp, Imp, Agg	Apa	Dysar	FTD/AD
6	83	Yes	20	Fullback	–	–	Head-to-player collision with LOC (1)	–	Mother (AD)	77	6	Agg, Dis, M	Yes	–	Agg, dis	–	Park, Trem, G	AD and PD
7	72	–	30	Centre-half	–	–	–	‘Blackouts’ since age 19 controlled with phenobarbital	–	58	14	M	Yes	–	Agg, Dis, Exp, Imp, Par	Apa, Dep	PI	AD
8	87	–	25	Wing-half	–	–	–	–	–	77	10	M	Yes	–	–	–	Park, Dysph	AD
9	92	–	25	Wing-half	–	–	–	–	–	78	14	M	Yes	–	Exp, Imp	Apa	–	AD
10	75	–	36	Winger	–	–	–	–	–	66	9	M	Yes	–	Agg, Exp, Imp,	Apa	PI, Dysar	AD and VasD
11	72	–	20	Centre-half	–	–	–	–	–	67	11	M	Yes	–	Agg, Imp	Apa	Park	AD
12	73	–	18	Centre-forward	Amateur boxing	Yes	–	–	Father (AD)	66	7	Dep, Dis	Yes	–	Agg, Exp, Imp, Par	Apa	–	VasD
13	65	–	24	Winger	–	–	–	–	–	40	15	Exp, M	Yes	–	Exp, Imp	Apa, Dep	Park, Trem, PI	AD and PD
14	66	–	30	Centre-half	–	–	‘Dazed and briefly drunk’ after heading the ball (occasional)	–	–	52	14	Dep, M	Yes	Yes	–	Apa, Dep	Park, Trem	AD

– absent, *AD* Alzheimer’s disease, *Anx* anxiety, *Agg* verbal and physical aggression, *Apa* apathy, *Aph* aphasia, *CBD* corticobasal degeneration, *CTE* chronic traumatic encephalopathy, *Dep* depression, *Dis* disinhibition, *Dysar* dysarthria, *Dysph* Dysphagia, *Exp* explosivity, *G* gait difficulties, *Imp* Impulsivity, GTC: Generalised tonic–clonic seizure, LBD: Lewy body disease, LOC: Loss of consciousness, M: Memory impairment, *Par* paranoia, *Park* Parkinsonism, *PD* Parkinson’s disease, *PI* postural instability with frequent falls, *TBI* traumatic brain injury, *Trem* tremor, *VasD* vascular dementia

^a^Progressive dementing illness accompanied by symptoms of memory impairment, executive dysfunction, disorientation, aphasia, and visuospatial impairment

All cases developed a progressive dementing illness in later life; ten of whom had coexisting motor impairments, including parkinsonism (*N* = 7), gait difficulties or postural instability with frequent falls (*N* = 6), and dysarthria (*N* = 3). Behavioural and mood changes were common features (*N* = 12). The average age at symptom onset was 63.6 years, and disease duration was 10 years. Twelve cases died from advanced neurodegenerative disease. Cases 5 and 10 died of myocardial infarction and ischaemic stroke, respectively. Substance or alcohol abuse and suicidal ideation were not reported in any cases. Twelve of the 14 cases had at least one CT or MR imaging of the brain following the onset of their neurological symptoms, and cortical atrophy was a consistent finding. Of the two earliest cases referred in the early 1980s, one had a normal air encephalogram and the other did not undergo neuroimaging. Cavum septi pellucidi was reported in one case (Case 11) on CT imaging of the brain performed 1 year after symptom onset, aged 68.

**Fig. 2:**
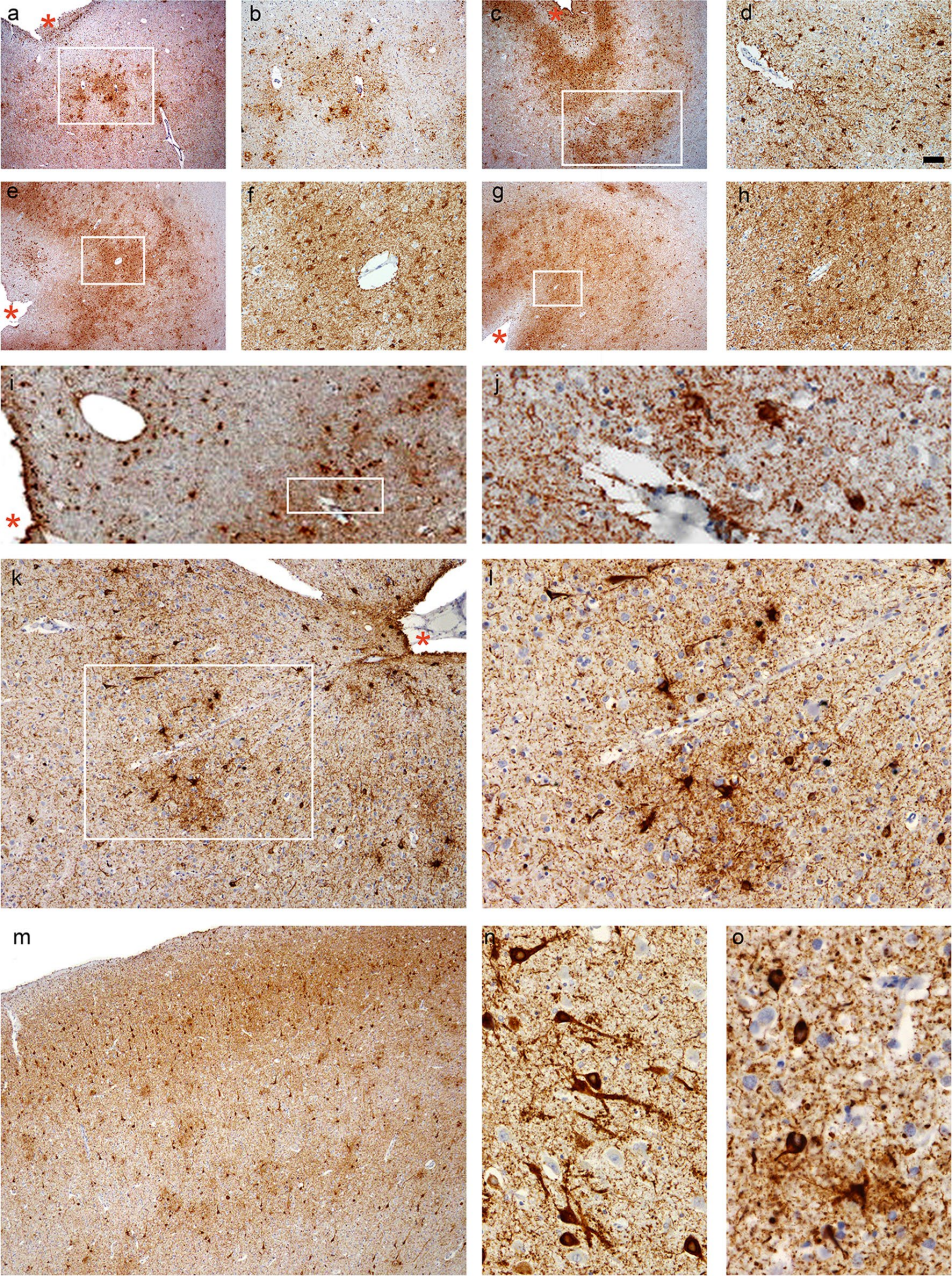
CTE pathology. **a**, **b** Case 1, parietal cortex, **c**, **d** Case 2, temporal cortex, **e**, **f** Case 5, temporal cortex, **g**, **h** Case 5, posterior frontal cortex (including the motor cortex), and **i**–**l** Case 6, temporal cortex. **b**, **d**, **f**, **h**, **j**, and **l** are images at high magnifications of the boxed regions on (**a**), (**c**), (**e**), (**g**), (**i**), and (**k**), respectively. **a**–**l** Patchy tau aggregates in neurons, astrocytes, and cell processes found preferentially at the depths of the cortical sulci with multiple perivascular foci. Cortical sulci are marked by asterisks. **m** Neuronal tau aggregates preferentially affecting superficial cortical layers (layers II–III) in CTE (Case 6, temporal cortex), which contrasts with the involvement of the deep cortical layers in Alzheimer’s disease (see Fig. 3). **n** Prominent proximal dendritic swellings in CA4 hippocampal subregion (Case 5). **o** Dot-like structures in the neuropils (Case 6, temporal cortex). All sections immunostained for AT8. *Bar* represents 100 µm in (**a**), (**c**), (**e**), (**g**), (**i**), and (**m**), 40 µm in (**b**), (**j**), and (**k**), 20 µm in (**d**), (**f**), (**h**), (**l**), and (**n**), and 10 µm in (**o**)

Neuropathological findings were available in six cases. While the mean age at symptom onset, age at death, and duration of football career did not differ between the post-mortem (Cases 1–6) and clinical (Cases 7–14) groups, the mean disease duration of the clinical group was relatively longer (11.8 years vs. 7.7 years, *P* = 0.01). Macroscopic brain examination revealed fenestration of the septum in all six cases and cavum septi pellucidi in one case (Case 1). Histological examination identified the pathognomonic features of CTE in four cases, fulfilling the mandatory diagnostic criteria of CTE [30], with Cases 2, 5, and 6 showing advanced CTE pathologies [32] (Fig. 2). Nevertheless, all six cases demonstrated some features supportive of CTE, including characteristic tau pathologies, dilatation of third ventricle, and septal abnormalities.

**Fig. 3:**
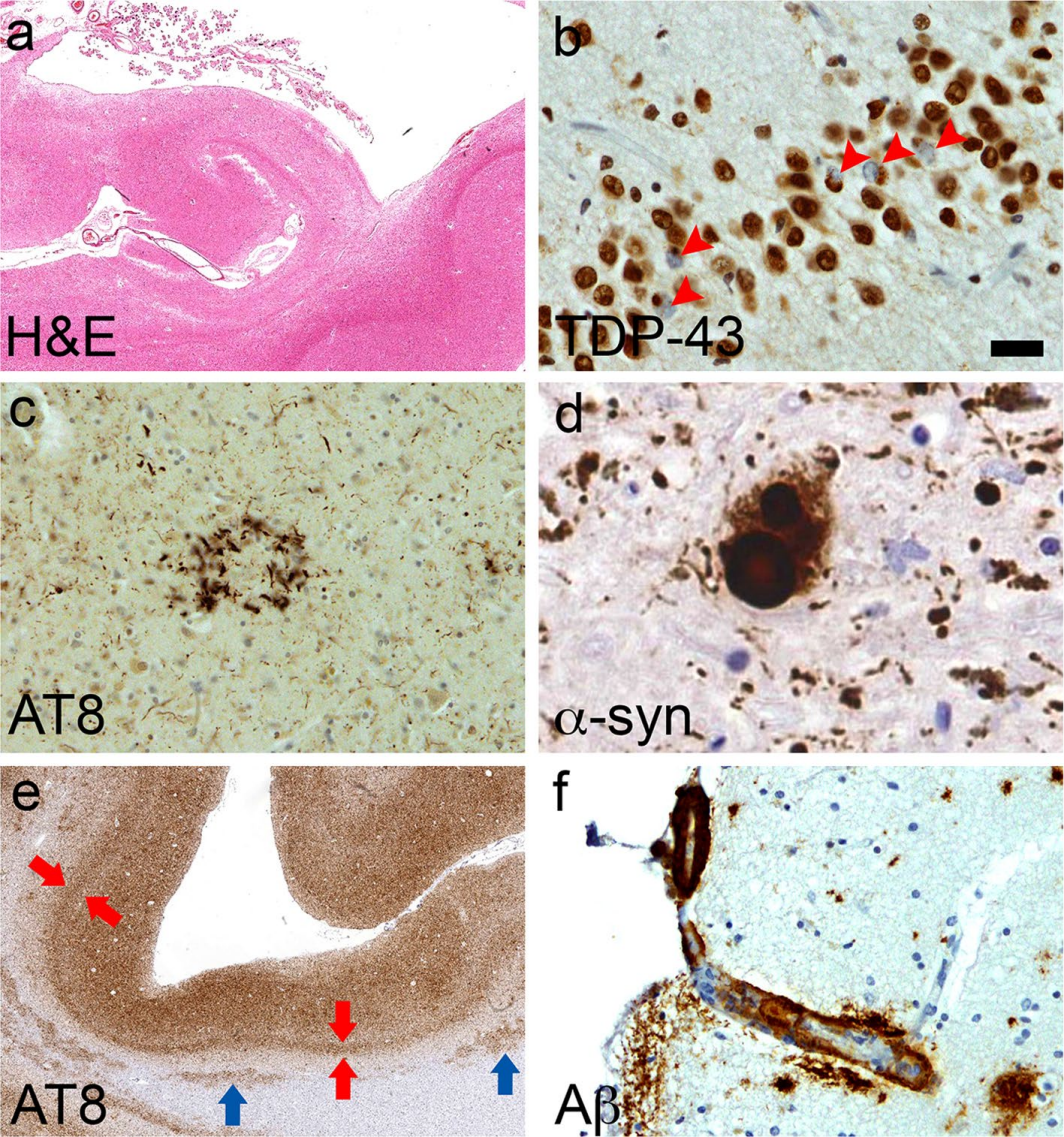
Mixed pathologies. **a** Hippocampal sclerosis (Case 2). **b** TDP-43-positive neuronal cytoplasmic inclusions in granule cells of the dentate gyrus (arrowheads; Case 2). **c** Astrocytic plaque in CBD (Case 1, temporal cortex). **d** Lewy body (Case 4, substantia nigra). **e** Uniform laminar distribution of Alzheimer-tau pathology which is particularly numerous in the deep cortical layers (*red arrows*, layer V), which contrasts with the patchy CTE-tau pathology observed in sulcal depths (see Fig. 2); tau-immunoreactive white matter astrocytes (*blue arrows*) are non-specific features of CTE (Case 3, frontal cortex). **f** Cerebral amyloid angiopathy of a cortical penetrating vessel (Case 1, frontal cortex). *Bar* represents 800 µm in (**a**), 600 µm in (**e**), 20 µm in (**c**) and (**f**), and 10 µm in (**b**) and (**d**)

All six cases had TDP-43 pathology with sparse to moderate TDP-43-positive dystrophic neurites and neuronal cytoplasmic inclusions (NCIs, Fig. 3). Occasional neuronal intranuclear inclusions (NIIs) were observed in Case 3. In Cases 2 and 3, TDP-43 pathology was restricted to the limbic regions, including the amygdala, entorhinal cortex, subiculum, and dentate gyrus, corresponding to Stage 3 of the staging scheme described for TDP-43 distribution in AD [18]. In the other four cases (Cases 1, 4, 5, and 6), TDP-43 pathology was more extensively distributed, involving regions, such as the striatum, substantia nigra, and cerebral cortices, corresponding to Stage 6 [18]. Features suggestive of diffuse axonal injury, such as axonal swellings (assessed by SMI31 immunohistochemistry), microglial nodules, or changes indicative of Wallerian degeneration (assessed by Iba-1 immunohistochemistry) were not observed in any of the six post-mortem cases [17].

Table 3 summarises the characteristic neuropathological findings in the six cases applying the NIND diagnostic criteria for CTE [30].

**Table 3:** Characteristic neuropathological features observed in the six footballers according to the preliminary NINDS criteria for the diagnosis of CTE [30]

Case	Tau pathologies (mandatory features)	Tau pathologies (Supportive features)					Non-tau pathologies (supportive features)		ARTAG with thorn-shaped astrocytes (non-diagnostic and non-supportive features)		
	perivascular, depths of cortical sulci, irregular pattern (neuronal and astrocytic)	Superficial cortical layers	CA2 (NFTs, PreTs, GTs), CA4 (proximal dendritic swellings)	Subcortical nuclei (neuronal and astrocytic)	Subpial and periventricular thorny astrocytes	Large grain-like and dot-like structures	Dilatation of 3rd ventricle, septal abnormalities	TDP-43	Subcortical white matter (patchy)	Mediobasal regions (subependymal, periventricular, perivascular)	Amygdala or hippocampus
1	Present (F, P)	Present	–	NA^a^	Present	NA^a^	Present	Present	–	Present	–
2	Present (F, T)	Present	–	Present	Present	Present	Present	Present	Present	Present	Present
3	–	–	–	Present	Present	–	Present	Present	Present	Present	Present
4	–	NA^b^	–	NA^b^	Present	NA^b^	Present	Present	Present	–	–
5	Present (F, T)	Present	Present	Present	Present	Present	Present	Present	Present	Present	Present
6	Present (T)	Present	Present	Present	Present	Present	Present	Present	Present	Present	–

– absent, *ARTAG* age-related tau astrogliopathy, *CA2 and CA4* hippocampal subregions, *F* frontal cortex, *P* parietal cortex, *T* temporal cortex, *TDP-43* transactive response DNA-binding protein 43 kDa pathology in hippocampus

^a^NA: not applicable or cannot comment in view of coexisting corticobasal degeneration-related tau pathology

^b^NA: not applicable or cannot comment in view of severe Alzheimer’s disease pathology

All cases had concomitant AD pathology (Fig. 3; Table 4) and some features of ARTAG (Table 3), which are currently not regarded as supportive of the diagnosis of CTE [30]. Histological features of CTE can be distinguished from coexisting AD pathology by patchy involvement of the superficial cortical layers, focal NFTs, and neuronal and astrocytic tau pathology adjacent to penetrating blood vessels with predilection of the depths of cortical sulci [4, 18, 30]. These specific histological features of CTE contrast with the uniform distribution of AD-tau pathology (neurofibrillary tangles) predominant in the deep cortical layers (Fig. 3e). Nevertheless, confluent tau pathology with high burden of AD-related changes in some cases can preclude the definitive diagnosis of CTE, as observed in Case 4 of the present series (Tables 3, 4). Cerebral amyloid angiopathy (CAA) and hippocampal sclerosis were observed in five and two cases, respectively. Argyrophilic grains were absent in the hippocampus and amygdala of all cases. Other concomitant diagnoses were CBD (Case 1) and LBD (Case 4). Case 3 had mild hyaline arteriosclerosis in the parietal and occipital cortices and basal ganglia. Case 6 had a small focal ischaemic infarct in the cingulate white matter and mild small vessel disease in the striatum.

**Table 4:** Neuropathological features of the six footballers included in this study

Case	Criteria-confirmed CTE [30]	Brain weight (g)	Septal abnormalities	Nigral cell loss	Hippocampal sclerosis	Braak NFT Stage	Thal Phase	‘ABC’ Score for AD (level)	CAA	TDP-43	Vascular pathology	Other pathological diagnosis
1	Yes	1250	CSP, F	Severe	–	IV	4	A3B2C2 (intermediate)	Moderate	Diffuse (Type A, Stage 6)	–	CBD
2	Yes	1150	F	–	Yes	IV	3	A2B2C2 (Intermediate)	–	Limbic (Type A, Stage 3)	–	–
3	No	1250	F	Mild	–	IV	4	A3B2C3 (intermediate)	Severe	Limbic (Type A, Stage 3)	Mild hyaline arteriosclerosis	–
4	No	1150	F	Severe	–	V	5	A3B3C3 (high)	Moderate	Diffuse (Type A, Stage 6)	–	LBD^a^
5	Yes	1120	F	Mild	Yes	IV	3	A2B2C2 (intermediate)	Moderate	Diffuse (Type A, Stage 6)	–	–
6	Yes	1500	F	Mild	–	IV	5	A3B2C2 (intermediate)	Moderate	Diffuse (Type A, Stage 6)	Small focal ischaemic infarct in cingulate white matter, mild SVD in striatum	–

– absent, *ABC score* ABC score for AD neuropathologic change (level) [34]—‘intermediate’ and ‘high’ are considered sufficient explanation for dementia, *AD* Alzheimer’s disease, *Braak NFT stage* Braak and Braak Neurofibrillary Tangle Stage (I–VI) [6], *CSP* cavum septi pellucidi, *CAA* cerebral amyloid angiopathy, *CBD* corticobasal degeneration, *CTE* chronic traumatic encephalopathy, *CTE criteria* preliminary NINDS criteria for the pathological diagnosis of CTE [30], *F* fenestration of septum, *LBD* Lewy body disease, SVD: Small vessel disease, *TDP-43* Transactive response DNA-binding protein, 43 kDa pathology in hippocampus: diffuse or limbic, recommended by the classification system of Mackenzie et al. for FDLD-TDP [27] and the staging scheme proposed by Josephs et al. for TDP-43 in AD [18], *Thal Phase* [41] thal Beta-amyloid Phase

^a^ *LBD* Braak stage 6 [7] and McKeith criteria for ‘diffuse neocortical’ category [33]

Table 4 summarises the main neuropathological diagnoses of these cases.

### Case summaries

#### Case 1

This man was a centre-forward. At 56, he became anxious and depressed. He gradually slowed up, his speech became slurred, and he was unsteady on his feet resulting in falls. Examination revealed rigidity and bradykinesia. At 60, he developed progressive non-fluent aphasia. He was irritable and aggressive. The clinical picture was a combination of FTD with parkinsonism.

The brain was mildly atrophic with dilatation of the fourth ventricle, cavum septi pellucidi with septal fenestration.

Histological examination of the neocortex showed superficial spongiosis and occasional swollen neurons. Tau immunohistochemistry revealed a combination of pre-tangles, neurofibrillary tangles (NFTs), threads, and astrocytic plaques, involving the neocortices, deep grey nuclei, hippocampal formation, brainstem, and the cerebellum, with threads and coiled bodies in the subcortical white matter, indicative of a diagnosis of CBD [11]. There was severe loss of pigmented neurons and gliosis in the substantia nigra.

The distribution of neuritic and Aβ-positive plaques and NFTs corresponded to ‘intermediate’ AD neuropathological changes (A3B2C2).

The superficial cortical layers displayed a high burden of neuronal and glial inclusions, positive for both 3-repeat and 4-repeat tau immunohistochemistry, supporting CTE-related pathology. Foci of perivascular astrocytic tangles (ATs) and NFTs in the sulcal depths of the frontal and parietal cortices were consistent with CTE [30]. There were occasional ghost tangles in CA1 and CA2.

#### Case 2

This man turned professional in his teens. He reported a head-to-head collision with loss of consciousness and skull fracture in a football match.

At 69, he had progressive episodic memory impairment and became eccentric. His MMSE score was 22/30 at age 71 and 17/30 at age 74. Past history included transient ischaemic attacks and hypertension. He developed impulsivity, grandiose ideation, and explosive rage attacks. In his last year, aged 78 with advanced dementia, a malignant abdominal mass was identified.

Examination of the brain showed moderate atrophy of the temporal lobes and hippocampi and fenestration of the septum pellucidum, with no evidence of metastatic tumour. Pigmentation in the substantia nigra was preserved.

The findings of multiple foci of tau-positive ATs and NFTs with more intense in the sulcal depths of the frontal and temporal cortices with a perivascular distribution were diagnostic of CTE [30]. In addition, there was widespread tau pathology supportive of CTE with neuronal and glial inclusions in the superficial cortical layers. CTE-tau pathology was widespread throughout the limbic structures, subcortical nuclei, and brainstem, in association with other supportive features (Table 3).

Neuritic and Aβ plaques were present in the neocortices, hippocampus, and striatum, corresponding to ‘intermediate’ AD neuropathological changes (A2B2C2). There was hippocampal sclerosis. Ghost tangles were observed in CA1, 3, and 4.

#### Case 3

This man played centre-half for over 20 years and was also an amateur cricketer. He reported a head-to-head collision with loss of consciousness during a football match.

At 66, he developed progressive episodic memory impairment, disorientation and became irritable and aggressive; gradually, he progressed to advanced dementia with occasional seizures. He died aged 74. Past history included hypertension and ischaemic heart disease.

Examination of the brain revealed mild focal atrophy of the cerebral cortex and hippocampi, marked dilatation of the third and lateral ventricles, and fenestration of the septum pellucidum. Tau immunohistochemistry demonstrated NFTs and threads in the deep cortical layers, hippocampal formation, amygdala, mammillary body and striatum, widespread neritic and Aβ mature, and diffuse plaques in the neocortices and hippocampus, consistent with ‘intermediate’ AD neuropathological change (A3B2C3). There was severe CAA in the neocortices, gliosis in the hippocampus (CA1, 4), and mild loss of pigmented neurons in the substantia nigra.

#### Case 4

This man was a dedicated amateur footballer and played either centre-half or centre-forward every season from the age of 10 for more than 20 years. He had one mid-air collision in a match which rendered him unconscious. He also boxed as an amateur but did not report any knockout or post-concussion symptoms.

He had a 5-year history of memory loss, irritability, aggressive behaviour, and visual hallucination which rapidly progressed to advanced dementia with parkinsonism, gait difficulties, and dysphagia. He died aged 60.

The brain was moderately atrophic especially in the medial temporal lobe and hippocampi. The septum pellucidum was fenestrated.

Tau immunohistochemistry revealed frequent NFTs and neuritic plaques with predilection for the deep cortical laminae. The extensive distribution of Aβ plaques and NFTs corresponded to a diagnosis of ‘high’ AD neuropathological change (A3B3C3).

Frequent α-synuclein immunoreactive Lewy bodies were observed in the cortices, cingulate gyrus, hippocampus, substantia nigra, and locus coeruleus with severe loss of pigmented neurons in the substantia nigra and locus coeruleus, compatible with advanced LBD (Braak stage 6 [7] and ‘diffuse neocortical’ category [33]).

#### Case 5

This man had a professional football career that spanned two decades. He reported one episode of head collision in a match which resulted in fractured jaw but no loss of consciousness.

At 63, he developed progressive memory loss, anomia, aggressive behaviour, and impulsivity. In his last year of life, he developed dysarthria and had moderately advanced dementia. He died at 72 of myocardial infarction.

The brain was moderately atrophic, predominantly affecting the medial temporal lobe and hippocampi, with dilatation of the lateral ventricles and fenestration of the septum pellucidum.

Tau immunohistochemistry showed foci of ATs, NFTs, and threads in the sulcal depths in the frontal and temporal cortices with a clear perivascular predilection, consistent with the diagnosis of CTE [30]. The predilection of tau aggregates in the superficial cortical layers and other supportive features of CTE were observed (Table 3) [32].

Neuritic and Aβ plaques were observed in the neocortices. The distribution of AD-related pathologies corresponded to ‘intermediate’ AD neuropathological change (A2B2C2). There was hippocampal sclerosis, frequent ghost tangles in CA1 and mild loss of pigmented neurons in the substantia nigra.

#### Case 6

This man reported one head-to-player collision with loss of consciousness while playing at an English football league match.

He had a 6-year history of memory impairment, intermittent disorientation, disinhibition, and aggressive behaviour, which progressed to dementia. Examination revealed hypomimia, hand tremor, limb rigidity, and bradykinesia. He died at 83.

Examination of the brain showed mild focal atrophy of the frontal and medial temporal lobes and hippocampi, dilatation of the lateral and third ventricles, and fenestration of the septum pellucidum. There was a small focal ischaemic infarct in the white matter underlying the cingulate cortex.

The widespread tau pathologies were consistent with a diagnosis of advanced CTE [32], which included tau-positive neuronal and astrocytic lesions with predilection for the sulcal depths of the temporal cortex in a perivascular distribution and other supportive features (Table 3) [30].

There were neuritic and Aβ plaques in the neocortices and hippocampus. The distribution of Aβ plaques and NFTs corresponded to a diagnosis of ‘intermediate’ AD neuropathological change (A3B2C2). Mild nigral cell loss was observed. There was mild small vessel disease in the striatum.

## Discussion

We report 14 retired footballers who developed dementia in later life. They were all skilled headers with half playing in positions that required frequent heading of the ball. Their playing career spanned two to three decades and most of them started training and heading the ball during childhood. The six post-mortem cases showed mixed pathologies, including criteria-defined CTE in four cases, AD and TDP-43 pathologies in six, CAA in five, and hippocampal sclerosis in two, and others had vascular pathology (*N* = 1), CBD (*N* = 1), and LBD (*N* = 1), and all would have contributed synergistically to the clinical manifestations. The frequency of CTE pathology in four of six cases in the present series represents a significant excess when compared with the 12% average background rate of incidental CTE pathology in elderly individuals with or without neurodegenerative disorders in our QSBB survey [25]. We hypothesize that CTE and, probably, AD and TDP-43 pathologies in these retired footballers are related to their past prolonged exposure to repetitive subconcussive head impacts from heading and head-to-player collisions.

Our four CTE cases add to the four case reports of footballers in the literature, three of whom had histological evidence of CTE (Table 5). Of the three cases with histological evidence of CTE, two presented with an AD-like dementia and died in their early 80s [13, 14] and another 29-year-old had amyotrophic lateral sclerosis (ALS) [31], substantiating the notion that playing football is a risk factor for CTE. The fourth footballer was an amateur and had AD pathology [4].

**Table 5:** Summary of clinical and pathological findings of footballers reported in the literature and present series

Case report	No. of cases	Age at death	Years playing football	Football playing position; level	History of concussion	Alcohol or substance abuse	Age at symptom onset	Disease duration	Presenting symptoms	Behavioural and mood changes	Other clinical features	Final clinical diagnosis	Septal abnormalities	Neuropathological diagnoses
McKee et al. [31]	1	29	26	NK but headed the ball frequently since age 5; semi-professional	NK	–	27	2	Limb weakness	–	–	ALS	NK	**CTE** MND TDP-43
Hales et al. [14]	1	80	16	Forward/striker; professional	NK	NK	70	10	Executive dysfunction and memory loss	Irritable, obsessive	Parkinsonism	AD	NK	**CTE** AD (mild) TDP-43 HS
Grinberg et al. [13]	1	83	21	Centre-back; professional	–	–	67	16	Memory loss	Shouting spells	Slow and abnormal gait	AD	C	**CTE** AD (Intermediate) TDP-43 HS SVD (Mild)
Bieniek et al. [4]	1	73	NK	NK; amateur	NK	Alcohol	66	7	Memory loss	NK	NK	AD	NK	AD
Ling et al. (present series)	6 (PM cases)	72 (mean)	25.7 (mean)	Centre-back or centre-forward (*N* = 3) but all headed the ball frequently; 5 professional, 1 committed amateur	In 5 footballers (only 1 episode in each)	–	64.3 (mean)	7.7 (mean)	Memory loss and/or behavioural changes	Yes (all)	Parkinsonism (3)	AD/FTD ± PD	F (6) C (1)	CTE (*N* = 4) AD (*N* = 6) TDP-43 (*N* = 6) CAA (*N* = 5) HS (*N* = 2) SVD (*N* = 1) CBD (*N* = 1) LBD (*N* = 1)

– absent, *AD* Alzheimer’s disease, *ALS* amyotrophic lateral sclerosis, *CAA* cerebral amyloid angiopathy, *CBD* corticobasal degeneration, *CTE* chronic traumatic encephalopathy, *F* fenestration, *HS* hippocampal sclerosis, *LBD* Lewy body disease, *PM* post-mortem, *SVD* small vessel disease, *TDP-43* transactive response DNA-binding protein 43 kDa, *NK* not known or not reported

There was no histological evidence of the previous diffuse axonal injury typically observed in acute traumatic brain injury, whereas all six of our post-mortem cases had septal fenestration and one also had cavum septi pellucidi. The finding of septal fenestration is supportive of their past histories of chronic repetitive head impacts from playing football [29]. The rate of septal abnormalities in our footballers is greater than the non-boxer general population, in whom septal fenestration and fenestration with cavum septi pellucidi were 6 and 3%, respectively, in autopsy [9]; while these macroscopic features were found in all 11 professional boxers in the Corsellis series, except in one case in whom cavum septi pellucidi was not observed, but ‘the septum was nevertheless fenestrated to destruction’ [9]. Concussion rate was limited in six of our 14 cases to one episode during their careers, which is a typical finding in professional footballers [19, 36] with one study reporting 74 episodes of concussion in 39 out of 72 male professional footballers [3]. Other potential repetitive head impacts outside the football field, including amateur boxing (*N* = 2), seizures (*N* = 2), and postural instability with frequent falls in later life (*N* = 4, Table 2), would have also contributed to the risk of CTE. The notion that prolonged exposure to repetitive subconcussive head impacts can lead to CTE is supported by the large Boston series, in which 16% of contact sport athletes and military veterans had CTE pathology but reported no concussion [32, 38].

Antemortem prediction of CTE pathology is difficult as all cases in the present series had a clinical presentation resembling either AD or FTD with the majority presenting in their 50s and 60s. In hindsight, motor impairments and early behavioural changes may serve as clinical pointers in the three cases with advanced CTE pathology (Cases 2, 5, and 6). Professional footballers also have an increased risk of developing ALS, but it was not a feature in our cases [8]. Mixed pathologies are the rule rather than exception in older individuals with dementia [1, 22]. For example, CTE, AD, TDP-43, and hippocampal sclerosis were also observed in the other two case reports of retired professional footballers [13, 14] (Table 5). We assume that the majority of our cases in the clinical group would have had mixed pathologies, including CTE. In CTE, AD and TDP-43 pathologies are common concomitant findings (Tables 3, 5) [32], and are increasingly considered as part of the CTE pathological entity, especially in older individuals [30] with the likelihood of Aβ deposition increased by APOE4 allele status [39]. The family history of dementia noted in our two oldest post-mortem cases (Cases 2, 6) may support a genetic predisposition. Unfortunately, frozen tissue and DNA were not available for genetic analysis in our cases.

This clinico-pathological series started as a surveillance that had spanned three decades, which was initiated and undertaken by a consultant psychiatrist (DDRW) with an interest in understanding the potential link between playing football and long-term neurodegenerative consequences. This descriptive study has a small number of cases without detailed psychometric, neuroimaging, or genetic data, yet its prospective collection of demographic data, playing and concussion history from close relatives, and regular surveillance from out-patient follow-up minimise case selection and recall bias in contrast to other post-mortem CTE series which relied on retrospective data collection only. Football is the most popular sport worldwide with over 250 million players at all levels. Although this study does not provide a firm causal relationship between CTE and exposure to repetitive head impacts from playing football, our findings support the pressing need to instigating large-scale studies to identify at risk groups of footballers, including age of exposure [37], which will justify for the implementation of protective strategies and education of current players. The significance of heading and the weight of the football remain elusive since the threshold of the impact force required to trigger the pathological process of CTE is currently unknown and repetitive head impacts in football are not limited to heading of the ball as footballers are also frequently exposed to head-to-player collisions [5]. All our cases were exposed to the heavier leather football used before the 1980s, which weighed 450 g and in wet condition became 25% heavier with the corresponding increased impact on contact with the head [2]. Nevertheless, lighter balls travel faster and may result in the same net force on head impact [19]. The assumption is that the lighter synthetic ball may also put modern footballers at risk of subconcussion and CTE is supported by the radiological findings of abnormal white matter microstructure in young footballers who headed the ball frequently [26] and the post-mortem finding of CTE in a 29-year-old footballer with ALS [31].

Future prospective longitudinal studies with radiological (including tau and amyloid PET scans and diffusion-tensor MRI), psychometric, biochemical, CSF, and genetic data in contemporary professional footballers with control group (e.g., athletes without increased risk of repetitive head impact), objective quantitative measure of head impact force, and clinical and pathological follow-up are required to confirm the potential causal relationship between CTE and exposure to repetitive head impacts from playing football.
